# High-affinity Cu(I) chelator PSP-2 as potential anti-angiogenic agent

**DOI:** 10.1038/s41598-019-50494-5

**Published:** 2019-10-01

**Authors:** Dorothea M. Heuberger, Shefali Harankhedkar, Thomas Morgan, Petra Wolint, Maurizio Calcagni, Barry Lai, Christoph J. Fahrni, Johanna Buschmann

**Affiliations:** 1 Institute of Intensive Care Medicine, University Hospital Zurich, Sternwartstrasse 14, 8091 Zurich, Switzerland; 2 School of Chemistry and Biochemistry and Petit Institute for Bioengineering and Bioscience, Georgia Institute of Technology, 901 Atlantic Drive, Atlanta, GA 30332-0400 USA; 3 Division of Plastic Surgery and Hand Surgery, University Hospital Zurich, Sternwartstrasse 14, 8091 Zurich, Switzerland; 4 Advanced Photon Source, X-ray Science Division, Argonne National Laboratory, Argonne, IL 60439 USA

**Keywords:** Drug development, Molecular medicine

## Abstract

Copper is an essential trace metal that has been implicated in angiogenesis, the formation of new blood vessels. As tumor growth relies on establishing a functional capillary network for blood supply, copper chelation therapy may hold promise as an anti-cancer strategy by suppressing angiogenesis. To test the anti-angiogenic effect of PSP-2, a recently developed high affinity Cu(I) chelator with low zeptomolar dissociation constant, we utilized the endothelial cancer cell line EAhy926 and assessed changes in cell migration, proliferation, and tube formation in Matrigel. In addition, sprouting was assessed by the chicken and sheep aortic ring assay, and vascular pattern formation was studied in the chorioallantoic membrane of chicken embryos (CAM assay). While incubation with PSP-2 resulted in selective depletion of cellular copper levels, cell migration was not affected and the proliferating activity was even slightly increased. Moreover, the endothelial tube formation assay revealed significant morphological changes in the presence of PSP-2, with thicker tubular walls and an overall decreased meshes area. Similarly, the aortic ring assay and CAM assay showed that PSP-2 evokes significantly longer sprouts with smaller angles at branching points. Altogether, PSP-2 exhibits significant bioactivity at concentrations as low as 5 μM, rendering it a promising anti-angiogenic agent. As EAhy926 cells exhibit both endothelial and tumorigenic properties, the anti-angiogenic effect of PSP-2 might potentially translate also into anti-cancer activity.

## Introduction

Copper is an important micronutrient involved in many fundamental biological processes^1^. An adequate supply of copper is not only critical for normal cell physiology but is also important in cancer progression^2^. Increased copper levels have been shown to stimulate angiogenesis, the formation of new blood vessels from existing vasculature, presumably by influencing the activity of proangiogenic factors, including VEGF, bFGF, TGFβ, and angiogenin^3^. Taking advantage of this effect, a range of biocompatible inorganic composite materials have been developed that promote vascularization in wound-healing by slow release of copper ions^4–6^. Conversely, copper depletion has emerged as a viable strategy to suppress vascularization in tumors^7,8^. For example, chelation therapy with penicillamine or tetrathiomolybdate has shown promising successes in reducing tumor growth, especially when combined with other anti-cancer regimens^9^. In addition, copper chelation has also been reported to induce anti-fibrotic and anti-inflammatory effects^8^.

The pro-angiogenic activity of copper cannot be reduced to a single target; rather, copper appears to play critical roles at multiple stages of angiogenesis^3^. At present, it remains unclear to what extent *in vivo* copper chelation affects the proliferation of endothelial cells or whether it suppresses blood vessel formation through a different mechanism. The mode of action might also differ between chelators, depending on the stability and redox potentials of the corresponding copper-complexes. For example, studies with tetrathiomolybdate suggest that its affinity is sufficient to sequester copper from cuproproteins such as ceruloplasmin, serum albumin, or metallothioneins. In addition, ternary cluster formation with Atox1, a copper-transporting metallochaperone, might block delivery to the trans-Golgi network where copper is inserted into nascent cuproproteins^10^.

We recently reported a membrane-permeant chelator, PSP-2, which binds Cu(I) with low zeptomolar dissociation constant (log*K* = 20, 25 °C) and exhibits negligible affinity towards other biologically relevant divalent metal cations, including Mn(II), Fe(II), and Zn(II)^11^. Despite the high Cu(I) affinity, PSP-2 revealed low acute toxicity in cell culture up to a concentration of 100 µM over a period of 24 hours. *In vitro* studies demonstrated that the chelator can intercept intracellular copper trafficking pathways, for example, by reversing copper-induced trafficking of the Menkes protein (ATP7A) or by blocking long-term potentiation of neurons in acute mouse hippocampal tissue slices^11^.

The favorable properties of PSP-2 as biological copper chelator thus prompted us to assess PSP-2 as a potential anti-angiogenic agent. To this end, we chose the human endothelial cancer cell line EAhy926 as a model system^12^, which was previously used in the context of endothelial cell damage^13,14^. As an initial *in vitro* assessment of anti-angiogenic activity^15^, we explored the effect of PSP-2 on cellular proliferation and endothelial tube formation. In addition, we utilized the chorioallantoic membrane of the chicken embryo (CAM assay)^16,17^ to assess the anti-angiogenic activity of PSP-2 *in ovo*. Furthermore, its effect on vascular sprouting was studied using the aortic ring assay^18^. The latter is an angiogenesis model where vascular sprouts are growing outside the aortic wall. In earlier studies, the assay has been employed to demonstrate the pro-angiogenic effect of copper, supplied in the form of CuSO_4_^19^, and to test the anti-angiogenic impact of drugs^20,21^.

Hence, we hypothesized that

(i) PSP-2 reduces EAhy926 cell proliferation,
(ii) PSP-2 changes the tube formation pattern in EAhy926 cells,
(iii) PSP-2 diminishes the sprouting in the aortic ring assay and
(iv) PSP-2 hinders normal angiogenesis in the CAM assay.

## Materials and Methods

### Chemicals

PSP-2 (1,2-bis(bis(dimethylphosphorothioylmethyl)phosphino)ethane) was synthesized as previously described^11^. Avastin was purchased from Kantonsapotheke Zürich (Bevacizumab 25 mg/ml, Kantonsapotheke Zürich, Switzerland).

### Cell culture

EAhy926 and murine NIH 3T3 cells were purchased from ATCC (LGC Standards GmbH, Wesel, Germany), and ATCC (Manassas, VA, USA), respectively. Passages P3-14 were used for all experiments. EAhy926 cells were grown in DMEM (Gibco, Thermo Fisher Scientific, Reinach, Switzerland) containing 10% fetal bovine serum at 37 °C, 5% CO_2_, 95% humidity. NIH 3T3 mouse fibroblasts were cultured in Dulbecco’s Modified Eagle’s Medium (DMEM), supplemented with 10% bovine calf serum and 1% penicillin-streptomycin, at 37 °C under an atmosphere of humidified air containing 5% CO_2_.

### Synchrotron X-ray fluorescence (SXRF) imaging

For SXRF imaging experiments, 3T3 mouse fibroblasts were grown on 5 × 5 mm silicon nitride windows (Silson Ltd., UK), pre-coated with 0.2 mg/mL solution of Human Fibronectin (Corning Cat. No. 354008). To increase copper levels above the limit of detection, cells were cultured in media supplemented with 50 µM CuCl_2_ which was removed prior to incubation with regular media containing 50 µM PSP-2 for 24 hours. Cells were then washed with PBS, fixed with a mixture of 3% paraformaldehyde and 1.5% glutaraldehyde in PBS for 20 min, washed again with PBS. The windows were air-dried overnight at room temperature by propping up on a rubber grid-mat placed in a covered sterile cell culture dish. SXRF imaging was performed at the 2-ID-D beamline of the Advanced Photon Source (Argonne National Laboratory, Argonne, USA). Cells were kept at room temperature under a He atmosphere and raster scanned through the incident beam with 10 keV excitation, which was focused to a spot size of 0.3 × 0.3 µm^2^ using a Fresnel zone plate^22^. Emission spectra were collected with a single-element fluorescence detector positioned at a 90 degree angle relative to the excitation beam, and elemental maps were generated by Gaussian fitting of the raw emission spectra using MAPS software^23^. Quantitative elemental densities were obtained by comparing the emission intensities from the sample to those of a thin film standard (AXO Dresden, Germany).

### Elemental quantification by total reflection x-ray fluorescence analysis (TRXF)

EAhy926 cells were cultured in DMEM supplemented with 10% fetal bovine serum and 1% penicillin-streptomycin at 37 °C under an atmosphere of humidified air containing 5% CO_2_. After growing cells in media supplemented with 50 µM CuCl_2_ for 24 h, the medium was replaced with regular medium with or without 50 µM PSP-2 and cells were incubated for another 24 h. Cells were then washed with PBS and harvested by trypsinization with trypsin-EDTA (1 mL). The cell count was determined with a hemocytometer, and cells were pelleted by centrifugation at 5000 rpm at 4 °C for 5 min. For the determination of total content of copper, the cell pellets were disrupted using 200 µL of concentrated nitric acid and digested for 48 h at room temperature. The analysis by TRXF was performed using a Bruker S2 PICOFOX spectrometer and the digested samples were standardized by addition of 1 µg/mL of Ga(III).

### Wound closure assay

EAhy926 cells were seeded into a non-coated 12 well plate (Techno Plastic Products AG, Trasadingen, Switzerland) in full media and allowed to reach confluency. Cells were scratched with a 10 µL tip (Starlab Group, Hamburg, Germany), washed three times with PBS before medium containing DMSO without and with PSP-2 at a final concentration of 5 µM was added. The wounded area was imaged at 20x magnification (Axiovert-10, Carl Zeiss AG) and analyzed at 0 h and 24 h post scratching using ImageJ (v1.47t; NIH, Bethesda, MA, USA). The size of the wound gap was measured at 3 random region of interests (ROI). Quantitative analysis of cell migration was performed based on the average wound gap from those ROIs, and the percentage of change was calculated using Eq. 1:

1 $$ \% \,change=\frac{({\rm{average}}\,{\rm{space}}\,{\rm{at}}\,{\rm{time}}\,{\rm{0}})\,-\,({\rm{average}}\,{\rm{space}}\,{\rm{at}}\,{\rm{time}}\,16\,{\rm{h}})}{({\rm{average}}\,{\rm{space}}\,{\rm{at}}\,{\rm{time}}\,0\,{\rm{h}})}\times \,100 \% $$

### CCK-8 proliferation assay

EAhy926 cells were seeded into a non-coated 96 well plate (Techno Plastic Products AG, Trasadingen, Switzerland) in full media. After cells reached confluency, the growth medium was replaced with media containing DMSO ± PSP-2 at final concentrations of 5, 10, and 20 µM, and the cells were incubated for another 24 h. CCK-8 reagent (Sigma, Buchs SG, Switzerland) was added to the media one hour before the viability was assessed by measuring the OD at 450 nm.

### Tube formation assay

EAhy926 cells were seeded into a 6-well plate and cultured in full media until they reached confluency. The cells were serum starved for one hour, washed with PBS, and harvested. Matrigel (Corning Matrigel Growth Factor Reduced Basement Membrane Matrix; Corning, NY14831, USA) was diluted 1:1 with serum-free media and prewarmed at 37 °C in a 96 well plate. After seeding with approximately 85000 cells in serum free media on the Matrigel, the cells were incubated at 37 °C for 24 h in medium with DMSO ± PSP-2 at a final concentration of 5 µM. Two to three images per well were captured at 10x magnification, using a phase contrast inverted microscope (Axiovert-10, Carl Zeiss AG, Feldbach, Switzerland), equipped with a digital camera. Images were analyzed using the Angiogenesis Analyzer, a plugin developed for the ImageJ (Version 1.47t; NIH, Bethesda, MA, USA).[ref]

### Aortic ring assay

(A) *Chicken:* Fertilized Lohman white LSL chicken eggs were purchased from Animalco AG, Switzerland, and incubated at 37 °C and 65% relative humidity in an incubator for 14 days. For experiments in chicken embryos until embryonic day 14 no IACUC approval is required according to Swiss animal care guidelines (TSchV, Art. 112). On incubation day 14, the aorta was carefully extracted according to established protocols^24^. Briefly, chick embryo aortic arches were excised after exposure of the chicken heart. Excess tissue around the aortic arches was removed and 2–3 rings per arch were achieved by cutting the arches perpendicularly. Aortic rings were imbedded in Matrigel and exposed to 3 groups of culture media; DMEM with DMSO, DMSO + PSP-2, or avastin, a non-copper based anti-angiogenic drug (positive control). For each of the 3 groups, 10 rings were used. (*B*) *Sheep:* For the sheep aortic ring assay, sheep aorta was dissected in disks of 5 mm in diameter. For experiments with sheep aorta from dead sheep, no IACUC approval is required according to Swiss animal care guidelines (TSchV). The disks were subsequently incubated in Matrigel and exposed to 2 groups of culture media; DMEM with either DMSO or DMSO + PSP-2. For each group, n = 5 rings were used. For quantitative assessment of morphological differences upon PSP-2 treatment, the number of sprouts within a defined region of interest (ROI) were counted (n = 7) and the branch lengths between two junctions were measured based on relative length units, with n = 158 and n = 190 for DMSO and DMSO + PSP-2, respectively. Angles between branches were assessed within the same ROI, with n = 94 and n = 122 for DMSO and DMSO + PSP-2, respectively. All quantitative readouts were evaluated with Synedra View 18.0.0.7 software.

### CAM assay

Fertilized Lohman white LSL chicken eggs were purchased from Animalco AG, Switzerland, and incubated at 37 °C and 65% relative humidity in an incubator for 3.5 days. For experiments in chicken embryos until embryonic day 14 no IACUC approval is required according to Swiss animal care guidelines (TSchV, Art. 112). Then, using a drill, a window was created in the eggshell after removing 2 mL of albumen^25^. The window was covered with a petri dish and incubated at 37 °C for another 3.5 days as described earlier^26^. On incubation day (ID) 7, a 5 mM stock solution of PSP-2 in DMSO was diluted into incubation buffer to a final concentration of 5 µM, and 100 uL of this solution was dripped onto the CAM surface with a silicone ring of 1 cm in diameter, confining the area of spreading liquid (control: diluted DMSO without PSP-2). On ID 8, another 100 µL of DMSO ± 5 µM PSP-2 were applied. On ID 14, at the end of the experiment, images were acquired of the whole ring. The sample size for each group was n = 4. For quantitative assessment, the lengths of the branches were determined based on relative length units using Synedra View 18.0.0.7 software, with n = 131 and n = 86 for DMSO and DMSO + PSP-2, respectively. Angles between branches were analyzed based on n = 91 and n = 36 for DMSO and DMSO + PSP-2, respectively.

### Statistical analysis

Data were analysed with StatView 5.0.1 and SPSS Statistics Version 25. Data were checked for normal distribution with Kolmogorov-Smirnow and Shapiro-Wilk tests. If data were not normally distributed, non-parametric tests, such as Kruskal Wallis test for more than 2 groups or Mann Whitney test for 2 groups, were conducted. For normal distribution, parametric one-way analysis of variance (one-way ANOVA) was conducted for more than 2 groups. Pairwise comparison probabilities (p values) were calculated using the Fisher’s PLSD. P values less than 0.05 were considered significant. Unpaired t tests were conducted for the comparison of 2 groups. The p values were indicated for pairs that were significantly different. Values were expressed as means ± standard deviations.

All methods were carried out in accordance with the relevant Swiss animal care guidelines and regulations.

## Results

### PSP-2 reduces total cellular copper without altering zinc or iron levels

**Figure 1:**
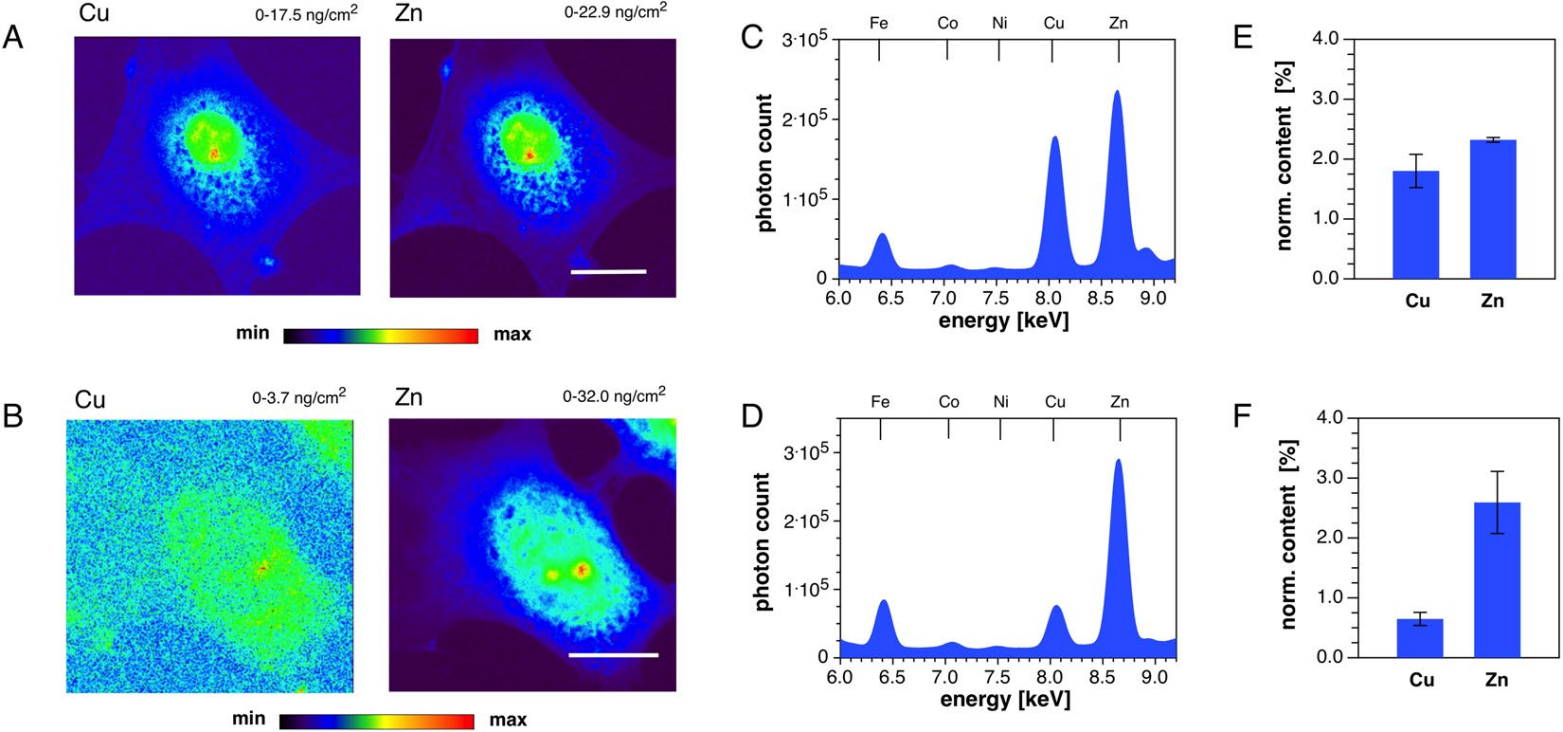
SXRF elemental analysis. SXRF analysis of 3T3 mouse fibroblasts grown in medium supplemented with CuCl_2_ (50 µM) before (A,C,E) and after (B,D,F) treatment with the Cu(I)-selective high-affinity chelator PSP-2 (50 µM). (**A**,**B**) False-color elemental density maps for Cu and Zn. The minimum to maximum density range is indicated at the top of each micrograph. Scale bar: 10 µm. (**C**,**D**) X-ray fluorescence spectrum showing the emission bands of Fe, Co, Ni, Cu, Zn. (**E**,**F**) Total cellular content of copper and zinc normalized to total sulfur to account for differences in cell size (n = 3).

In earlier studies, we demonstrated that PSP-2 can readily traverse cell membranes and intercept copper-dependent intracellular pathways^11,27^. To investigate whether the effects of PSP-2 actually correspond to selective removal of cellular copper, we visualized changes in trace metal content by synchrotron x-ray fluorescence (SXRF) microscopy using 3T3 mouse fibroblasts as a model cell line (Fig. 1). Because endogenous copper levels are low, often residing at the limit of detection for single cell SXRF analysis, cells were grown in media supplemented with 50 µM CuCl_2_. As evident from the false-color density maps, zinc and copper showed similar distribution patterns with a pronounced nuclear localization (Fig. 1A). Upon incubation with 50 µM PSP-2 for 24 h, copper levels decreased to approach the limit of detection, while zinc levels remained unaffected (Fig. 1B). A comparison of the corresponding x-ray fluorescence emission spectra, averaged over the entire cell area, best illustrate the effect of PSP-2 (Fig. 1C,D). While the copper signal dramatically decreased, iron and zinc remained essentially unaltered. Further quantification relative to a NIST standard yielded an approximately 3-fold decrease in the cellular copper content whereas the average zinc content remained unchanged upon PSP-2 treatment (Fig. E,F). To account for differences in cell size, the integrated copper and zinc densities were normalized to the sulfur content of each cell.

**Figure 2:**
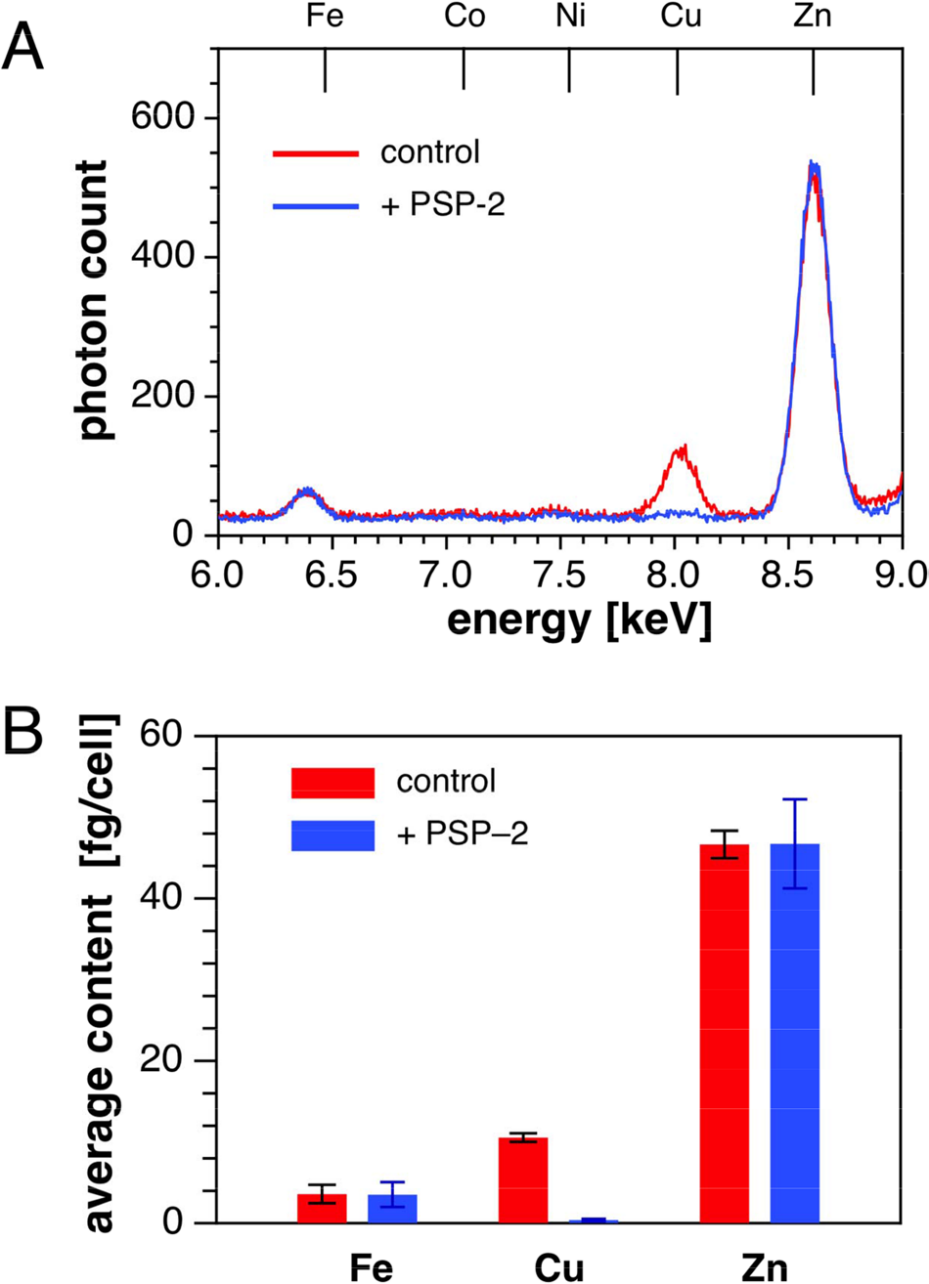
TRXF elemental analysis. TRXF elemental analysis of EAhy926 endothelial cells grown in medium supplemented with CuCl_2_ (50 µM) and treated with or without PSP-2. (**A**) Averaged x-ray fluorescence emission spectra derived from cells that were incubated for 24 hours with full medium in the absence (control) or presence of 50 µM PSP-2 (n = 5, averaged x-ray emission spectrum of 10^5^ cells). (**B**) Quantification of the x-ray emission using Ga(III) as internal standard (n = 5).

To test the effect of PSP-2 with cells relevant to angiogenesis, we determined changes of the average elemental content using the human umbilical vein endothelial cancer cell line EAhy926. Elemental analysis of the average copper content by total reflection x-ray fluorescence analysis (TRXF) revealed a dramatic effect after incubating with PSP-2 for 24 h with a greater than 20-fold decrease compared to control (Fig. 2). In agreement with above SXRF data, the relative x-ray emission signals for zinc and iron remained unaffected by PSP-2 (Fig. 2A). Altogether, these data demonstrate that PSP-2 is able to selectively reduce copper levels within the complex chemical environment of a live cell.

### Wound closure and cellular proliferation assay with EAhy926 endothelial cells

**Figure 3:**
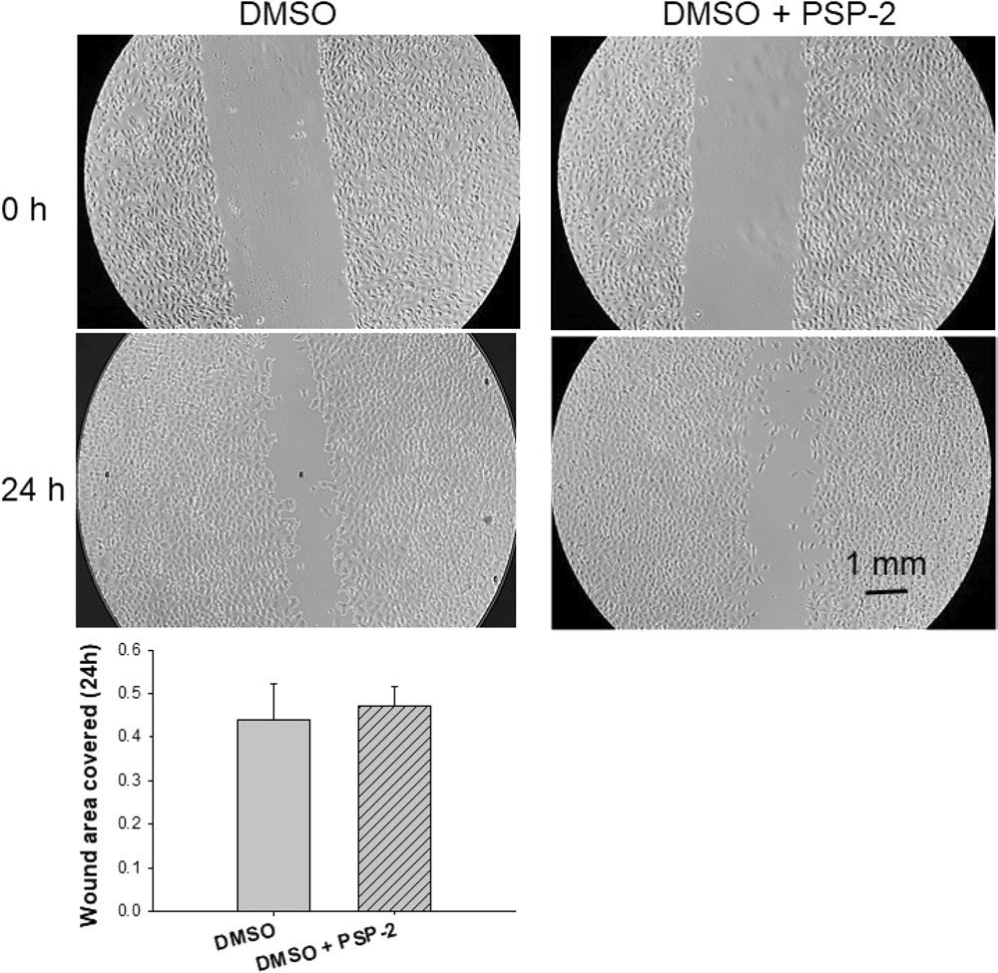
Wound closure assay with EAhy926 cells. A confluent monolayer of EAhy926 cells was scratched and the wound closure was assessed after 24 hours of migration towards the scratched area (covered area as fraction of whole area). PSP-2 was diluted into incubation buffer from a 5 mM stock solution in DMSO to yield a final concentration of 5 µM. For control experiments, an equivalent amount of DMSO without PSP-2 was added. Sample size n = 9. The p value between the two groups was 0.6404 (STUDENT’s t test).

The stimulating effect of copper on endothelial cell proliferation is well documented^28^. To explore whether PSP-2-mediated copper sequestration might affect wound healing, we performed an *in vitro* scratch assay with EAhy926 cells. In this assay, cell migration is monitored over time after creating a “scratch” in a confluent cell monolayer^29,30^. As evident from Fig. 3, after 24 hours we observed no statistically significant difference in cell migration between cells exposed to 5 µM PSP-2, supplied from a 5 mM stock solution in DMSO, and cells treated with DMSO at the same concentration after a 1:1000 dilution.

**Figure 4:**
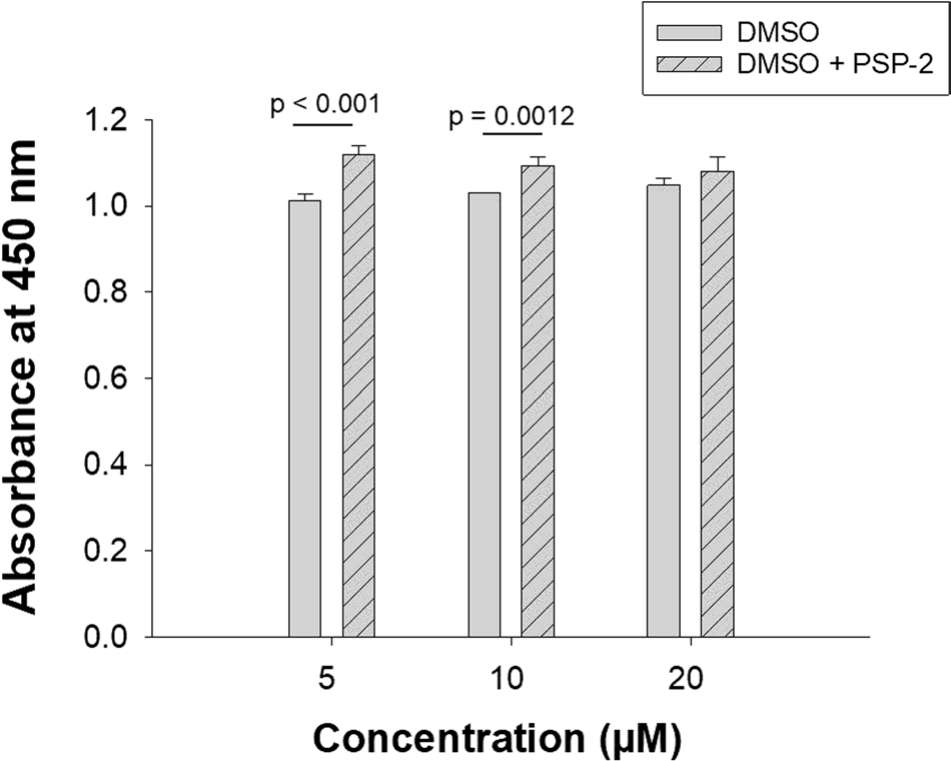
Effect of PSP-2 on the proliferation of EAhy926 cells (CCK-8 assay). Absorbance of the CCK-8 reagent at 450 nm of EAhy926 cells after 24 h exposure to PSP-2 at different concentrations, supplied from a DMSO stock solution. Control cells were treated with an equivalent amount of DMSO. Legend: DMSO: cells treated with DMSO only; DMSO + PSP-2: cells treated with equal amounts of DMSO and PSP-2. Sample size n = 9, analysis by (STUDENT’s t test).

In order to assess the effect of PSP-2 on cellular proliferation, we performed a CCK-8 viability assay. While at low concentrations of PSP-2 the proliferation of EAhy926 cells was slightly enhanced, we observed no significant difference at 20 µM PSP-compared to untreated control cells (Fig. 4). Although small, the increases in cellular proliferation at 5 and 10 µM ligand concentrations were statistically significant.

### Tube formation assay with EAhy926 cells

**Figure 5:**
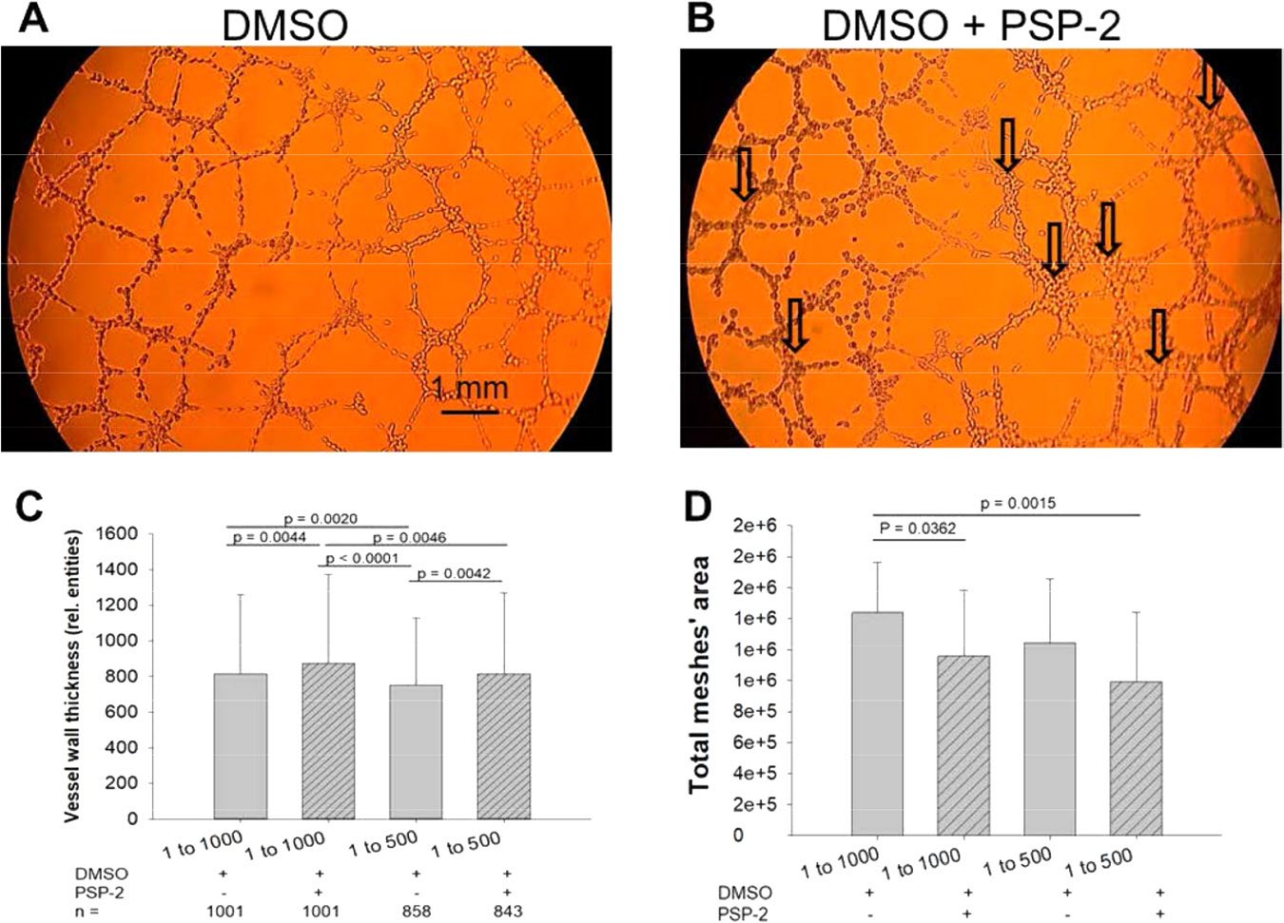
Tube formation assay with EAhy926 cells. The effect of PSP-2 on tube formation (**A**) is expressed by wall thickening (**B**, arrows, and **C** (Kruskal Wallis test)) and by total meshes area (**D**, 1-way ANOVA). PSP-2 was diluted into incubation buffer from a 5 mM stock solution in DMSO to yield the indicated final concentrations. For control experiments, an equivalent amount of DMSO without PSP-2 was added.

When plated on Matrigel, endothelial cells form capillary-like mesh structures within a few hours, thus offering a convenient model for the rapid screening of angiogenesis inhibitors^31^. In the presence of 5 or 10 µM PSP-2, tube formation resulted in a visual change in morphology with a significant increase in wall thickness as well as a reduction in the number of tubular walls (Fig. 5, and Supporting Information, SI Fig. 1). In addition, PSP-2 treatment reduced the total meshes area at both incubation concentrations.

### Outgrowth of endothelial cells using the chicken aortic ring assay

**Figure 6:**
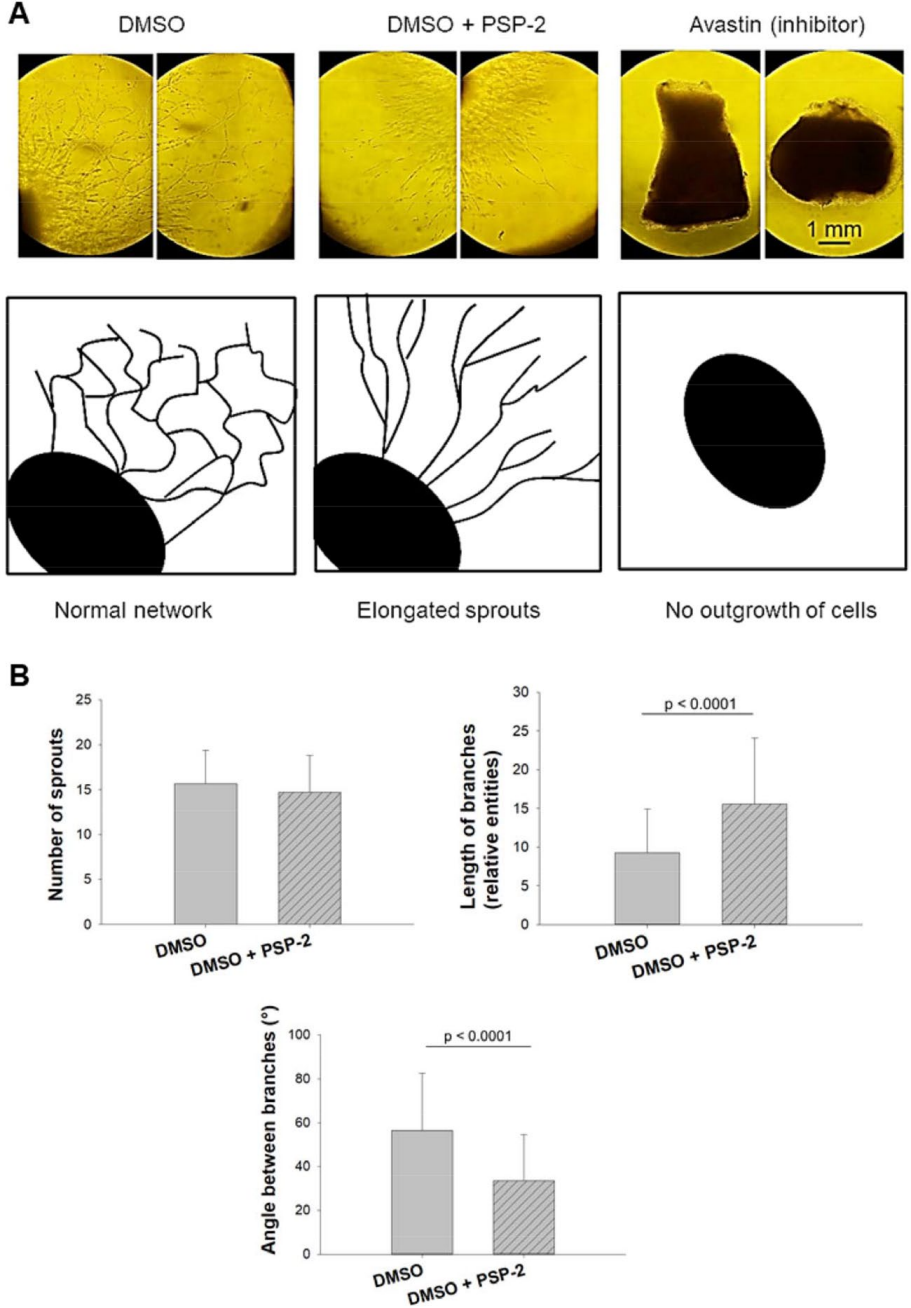
Aortic ring assay. Qualitative assessment of outgrowth endothelial cells (**A**). DMSO treatment has no effect on network formation (left), while DMSO + 5 µM PSP-2 leads to fewer branches but longer sprouts in the chicken aortic ring assay (middle). With avastin, an anti-angiogenic agent, no sprouting was observed (right). Prevalent outgrowth structures are schematically shown below. Quantitative readouts (**B**), with number of sprouts within a region of interest (ROI); length of branches between two junctions and angle between branches, analyzed by Mann Whitney test. For comparison, sheep aortic ring assay was performed, showing similar effects of outgrowth patterns as found in the chicken aortic ring assay (see Supporting Information, SI Fig. 2).

Contrary to the effect of avastin, a potent inhibitor of angiogenesis, the outgrowth of endothelial cells in the chicken aortic ring assay was not compromised in the presence of PSP-2; however, the outgrowth pattern was distinctly different compared to control. While in the presence of DMSO alone a regular network was formed, the presence of PSP-2 led to a network with fewer branches and overall elongated vessels, which assumed a parallel alignment to each other (Fig. 6).

### CAM assay

**Figure 7:**
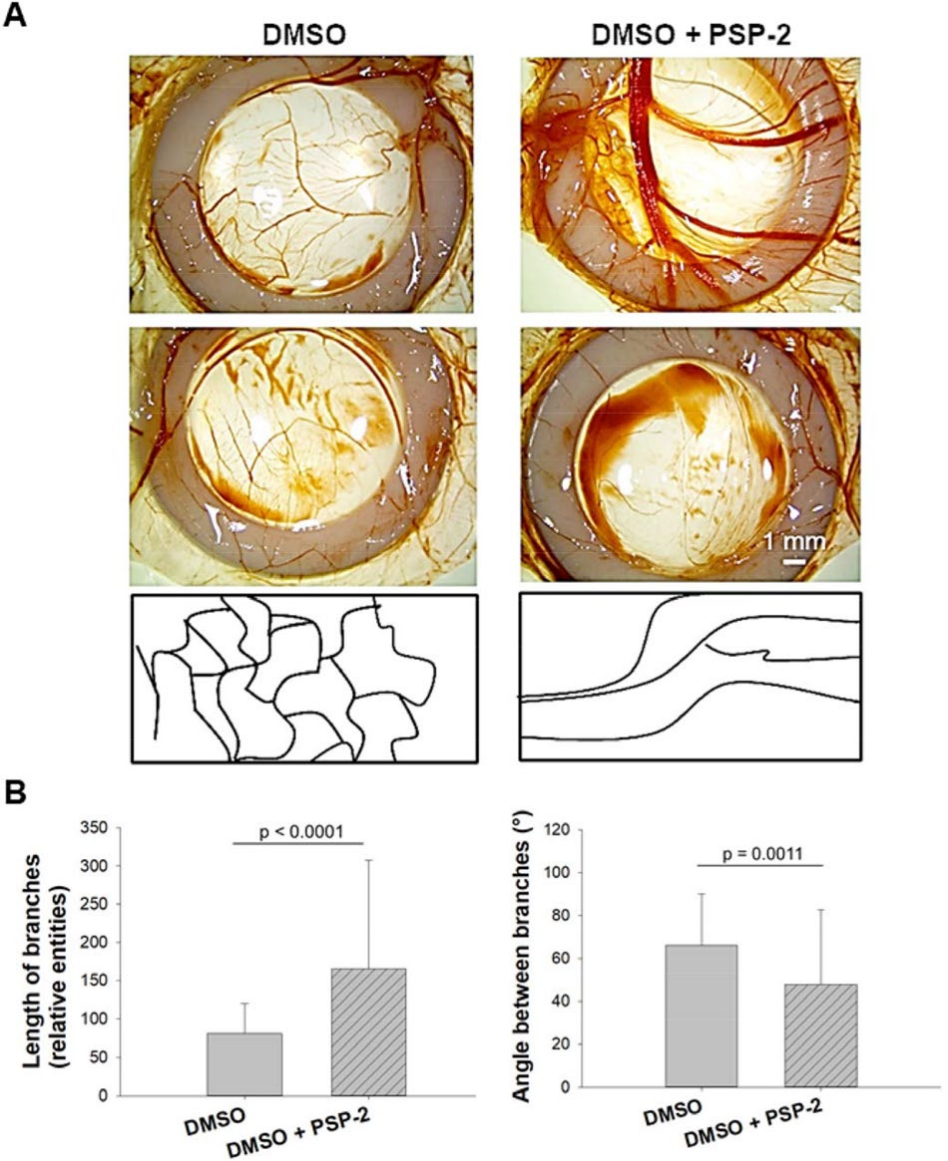
Angiogenesis assessed by the CAM assay. A solution of 200 µL of DMSO ± PSP-2, which was diluted in incubation buffer to a final concentration of 5 µM PSP-2, was dripped onto the CAM surface, followed by a second application after 24 hours. Images were acquired 7 days after the first application (**A**). Quantitative assessment of the length of branches between two junctions and angle between branches (**B**), analyzed by Mann Whitney test.

The CAM assay has been frequently used to study pro- and anti-angiogenic effects evoked by drugs^32^ or biomaterials^33^ placed on the chorioallantoic membrane of living chicken embryo^34,35^. Moreover, vascularization can be assessed not only by histology *ex vivo* but also longitudinally at different time points by MRI *in ovo*^26^. Finally, the CAM assay is inexpensive and easy to execute. Application of PSP-2 in the CAM assay revealed a distinct change in the vascular pattern (Fig. 7). Major structural differences upon PSP-2 application were found in a reduced number of branches and in longish vessels compared to treatment with DMSO only, which was used as control.

## Discussion

In this study, we demonstrated that the high-affinity Cu(I)-chelator PSP-2 exhibits significant anti-angiogenic effects. Consistent with previous metal ion selectivity studies in aqueous buffer^11^, treatment of 3T3 cells with PSP-2 resulted only in a decrease of copper levels without affecting other biologically relevant transition metals such as iron and zinc (Fig. 1). Moreover, the subcellular distribution of zinc remained qualitatively unchanged as revealed by x-ray fluorescence microscopy (Fig. 1). Likewise, PSP-2 selectively removed copper from EAhy926 cells, which were chosen as a model cell line as it offers both endothelial as well as tumorigenic characteristics (Fig. 2).

Despite the ability to deplete total copper levels, PSP-2 did not reduce cellular proliferation and migration (Figs 3 and 4). At low incubation concentrations, cell proliferation even increased by approximately 10% in the presence of 5 μM PSP-2, and around 5% with 10 μM PSP-2. At a concentration of 20 μM PSP-2, however, there was no significant difference compared to untreated control cells (Fig. 4). These data contrast the effect of tetrathiomolybdate, which inhibited proliferation of human umbilical vein endothelial cells (HUVECs) already at a concentration as low as 2 µM^36^, or of other anti-angiogenic agents such as Sorafenib, which reduced proliferation of HUVECs by 40% at a concentration of 4 μM^37^. However, as cellular proliferation might be differently regulated in the EAhy926 cancer cell line, the HUVECs studies are not directly comparable.

The most striking anti-angiogenic effect of PSP-2 was observed during network formation of EAhy926 cells. In the presence of 5 μM PSP-2, we observed a pronounced wall thickening as well as a significant reduction of the total meshes area (Fig. 5). Similar effects were reported for tetrathiomolybdate^36^ as well as the anti-angiogenic agent arsenic oxide (As_2_O_3_)^37^. In the presence of PSP-2, vascular tube formation was disrupted in favor of cell accumulation at branching points to produce fewer but much thicker walls. The morphology of the networks formed in the presence of PSP-2 completely differed from the control. These results parallel earlier reports where copper chelation had no effect on endothelial process formation but resulted in almost complete inhibition of network formation^38^. As the tube formation assay is usually performed with HUVECs^39^ and includes different aspects of angiogenesis, such as endothelial cell adhesion, migration, alignment, and tube formation, it is noteworthy that PSP-2 affected tube formation of EAhy926 cells, which represent not only endothelial cell characteristics but also tumorigenic properties.

The *in vitro* anti-angiogenic activity of PSP-2 in the tube formation assay was further confirmed by two additional assays, the aortic ring assay as well as the CAM assay. Previous reports showed that bovine aortal cell migration was significantly increased in the presence of 1 μM CuSO_4_^19^, thus underscoring the role of copper in angiogenesis^3,40^. In the chicken aortic ring assay, 5 μM PSP-2 did not suppress outgrowth of endothelial cells as observed with the inhibitor avastin, which was used as positive control. However, the outgrowth pattern significantly changed to yield longer sprouts with fewer branching points (Fig. 6A). Quantitative readouts in the aortic ring assay could be assessed only beyond a cell-dense area of fibroblasts. Although the number of endothelial cell sprouts was very similar, the lengths of the branches were significantly higher in the samples that were incubated with PSP-2. Moreover, PSP-2 treatment led to significantly smaller angles between the branches (Fig. 6B).

A similar vessel pattern was found in the CAM assay, where PSP-2 suppressed branching, in favor of long, parallel arranged vessels within the silicone ring (Fig. 7A). Likewise, the length of the branches was significantly increased in the samples that were treated with PSP-2, with significantly smaller angles between the branches (Fig. 7B). Taken together, both assays confirmed the results found in the tube formation assay with EAhy926 cells. Endothelial cells from the chicken aorta as well as vessel sprouting on the chorioallantoic membrane (including primarily endothelial cells), were hindered to form regular tube-like structures.

## Conclusions

In this study, we showed that PSP-2, a high-affinity Cu(I) chelator with low zeptomolar dissociation constant, selectively reduces cellular copper levels and exhibits significant anti-angiogenic activity in the tube formation assay performed with EAhy926 endothelial cancer cells. Moreover, PSP-2 treatment resulted in significantly longer sprout branches with smaller angles in the chicken aortic ring assay. Finally, PSP-2 applied in the CAM assay significantly elongated vessel branches and reduced the angle between vessels. In light of the tumorigenic nature of EAhy926, PSP-2 might harbor not only anti-angiogenic, but also anti-tumorigenic activity, worthwhile of further exploration in an animal model. In addition, further research is needed to evaluate anti-cancer effects of PSP-2 in comparison to and in combination with other anti-cancer drugs.

## Supplementary information

Supplementary Information
