# Supplementary material for: High-affinity Cu(I) chelator PSP-2 as potential anti-angiogenic agent
Source: Sci Rep. 2019 Oct 1;9:14055. doi: 10.1038/s41598-019-50494-5 (PMC6773859; doi:10.1038/s41598-019-50494-5)
Supplement: Supplementary file 1 — Supplementary Information [file 41598_2019_50494_MOESM1_ESM.pdf]

Supporting Information

High-affinity Cu(I) chelator PSP-2 as potential anti-angiogenic agent

Dorothea M. Heuberger[1], Shefali Harankhedkar[2], Thomas Morgan[2], Petra Wolint[3], Maurizio Calcagni[3], Barry Lai[4], Christoph J. Fahrni[2]* and Johanna Buschmann[3]*

[1]Clinic of Intensive Care, University Hospital Zurich, Sternwartstrasse 14, 8091 Zurich, Switzerland.

[2]School of Chemistry and Biochemistry and Petit Institute for Bioengineering and Bioscience, Georgia Institute of Technology, 901 Atlantic Drive, Atlanta, GA, 30332-0400, USA.

[3]Division of Plastic Surgery and Hand Surgery, University Hospital Zurich, Sternwartstrasse 14, 8091 Zurich, Switzerland.

[4]Advanced Photon Source, X-ray Science Division, Argonne National Laboratory, Argonne, IL 60439, USA.

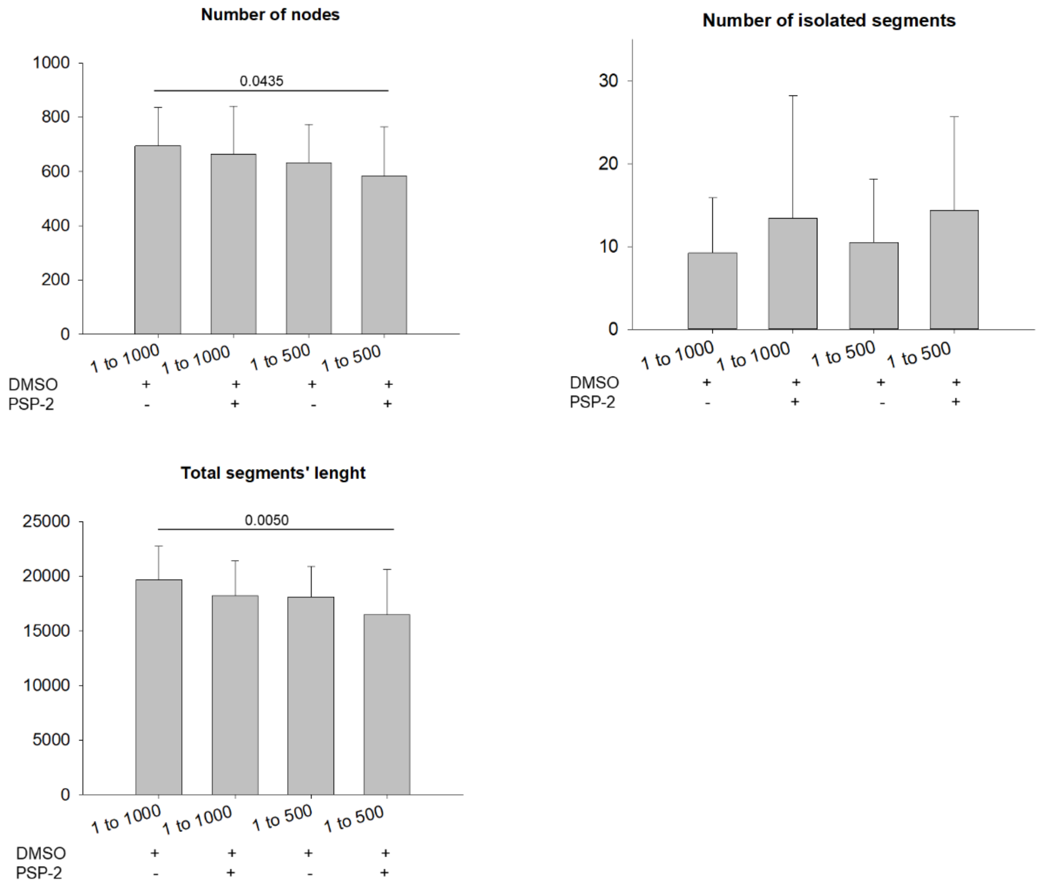

SI Figure 1 Further readouts of tube formation assay. Besides wall thickness and total meshes' area (**Figure 4**), further readouts from the tube formation assay were assessed for two concentrations of PSP-2, 5 and 10 µM, with dilutions of 1 to 1000 and 1 to 500, respectively, from a stock solution of 5 mM PSP-2: Number of nodes, number of isolated segments and total segments' length. *p* values for one-way ANOVA statistical analysis are given for pairs having a *p*-value < 0.05.

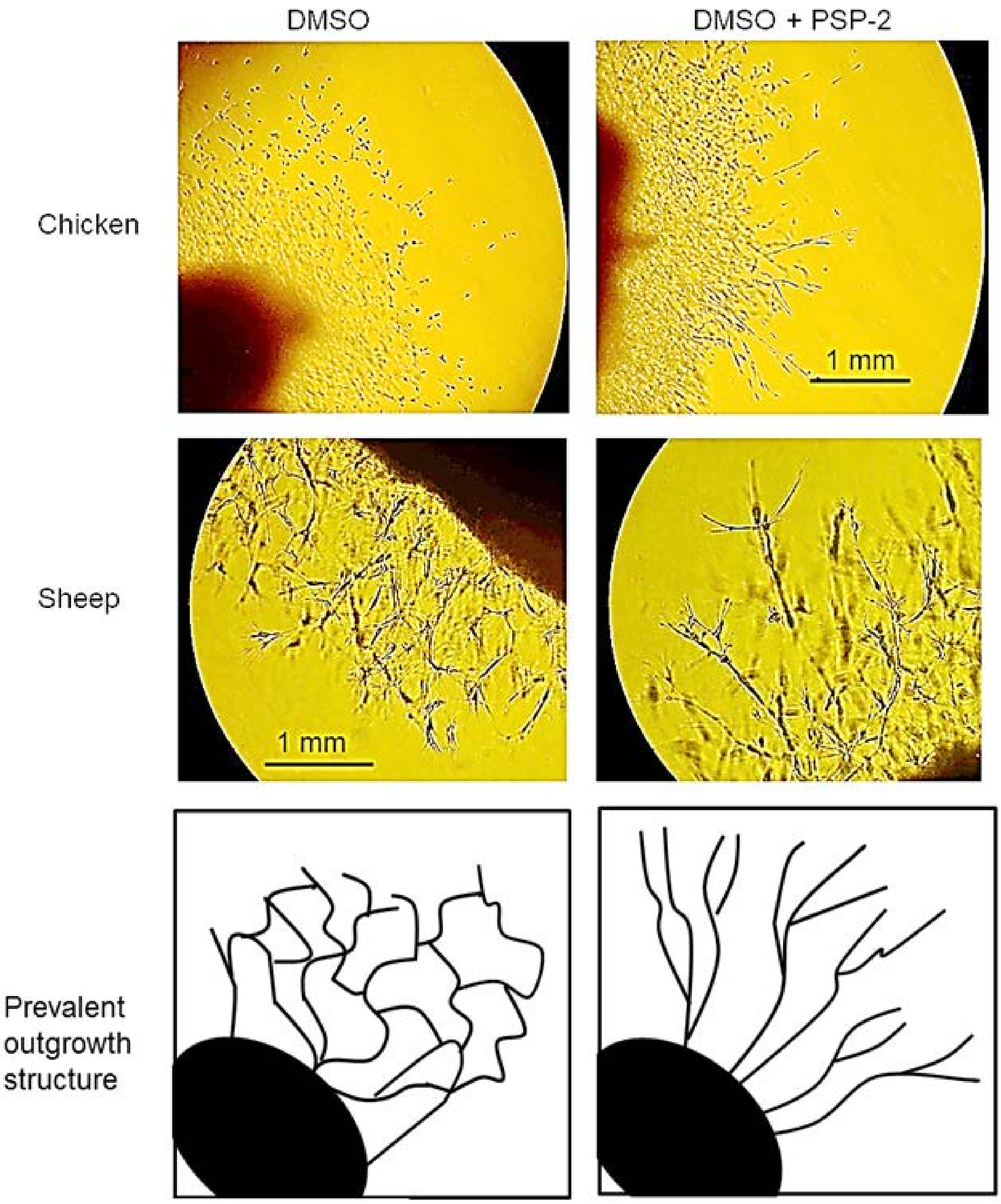

SI Figure 2: **Chicken and sheep aortic ring assays** with typical prevalent outgrowth structures. *Key*: DMSO + PSP-2 refers to DMSO with 5 µM PSP-2 after a 1:1000 dilution into incubation buffer.
